# Pharmacological and Clinical Efficacy of *Picrorhiza kurroa* and Its Secondary Metabolites: A Comprehensive Review

**DOI:** 10.3390/molecules27238316

**Published:** 2022-11-29

**Authors:** Tahani M. Almeleebia, Abdulrhman Alsayari, Shadma Wahab

**Affiliations:** 1 Department of Clinical Pharmacy, College of Pharmacy, King Khalid University, Abha 61421, Saudi Arabia; 2 Department of Pharmacognosy, College of Pharmacy, King Khalid University, Abha 61421, Saudi Arabia; 3 Complementary and Alternative Medicine Unit, College of Pharmacy, King Khalid University, Abha 61421, Saudi Arabia

**Keywords:** *Picrorhiza kurroa*, medicinal plants, natural product, phytochemical, biological activity, toxicity

## Abstract

Traditional remedies for the treatment of various ailments are gaining popularity. Traditionally, one of the most valuable therapeutic herbs has been *Picrorhiza kurroa* Royle ex Benth. Traditional and folk uses of *P. kurroa* include chronic constipation, skin-related problems, burning sensation, chronic reoccurring fever, jaundice, heart problems, breathing, digestion, allergy, tuberculosis, blood-related problems, prediabetes and obesity, laxative, cholagogue, and liver stimulatory. Phytoconstituents such as glycosides, alkaloids, cucurbitacins, iridoids, phenolics, and terpenes in *P. kurroa* have shown promising pharmacological potential. In order to uncover novel compounds that may cure chronic illnesses, such as cardiovascular, diabetes, cancer, respiratory, and hepatoprotective diseases, the screening of *P. kurroa* is essential. This study comprehensively evaluated the ethnopharmacological efficacy, phytochemistry, pharmacological activity, dose, and toxicity of *P. kurroa*. This review provides comprehensive insights into this traditional medication for future research and therapeutic application. The purpose of this review article was to determine the pharmacological effects of *P. kurroa* on a variety of disorders. *P. kurroa* may be a natural alternative to the standard treatment for eradicating newly evolving diseases. This study is intended as a resource for future fundamental and clinical investigations.

## 1. Introduction

The steadily growing life expectancy, contemporary lifestyles, and changing environmental circumstances are responsible for the rising prevalence of the processes leading to various illnesses. The death rate due to multiple ailments has increased worldwide. Over the years, it has become necessary to seek new treatments that may cure chronic diseases and other infectious disorders [1,2]. The primary focus of the study is to identify natural compounds with a lower potential for adverse effects than synthetic pharmaceuticals because of the significant adverse effects caused by synthetic medications [3].

*Picrorhiza* is derived from the Greek language, which means “bitter” (picros) and “roots” (rhiza). *Picrorhiza* is a genus comprising two species (*Picrorhiza scrophulariiflora* Pennell and *Picrorhiza kurroa* Royle ex Benth) of the family Plantaginaceae. Most species are found in nature at sites such as cliffs, crevices, and mountainsides. *Picrorhiza kurroa* Royle ex Benth is mainly found at an altitude of 3000–5000 m above sea level in the Himalayan ranges of Nepal, Pakistan, and India [4,5]. This plant grows on rocky slopes and organic and moist soils in the Himalayan region (Garhwal to Bhutan). It is also found in northern Burma, western China, and southeastern Tibet [5]. It is also grown in the central and eastern regions but is abundant in the northwestern region [4]. It is a well-established herbal medicine for many ailments, ranging from dyspepsia to hepatitis [6]. Traditional and modern medicine have used bitter extracts from the dried rhizomes of this plant as a purgative, brain tonic, stomachic, and antiperiodic to treat dyspepsia [7,8,9]. Many traditional herbs are known for their cooling, heating, or neutral properties. If the heat is reduced by a herb, it becomes known for its cooling properties. *P. kurroa* has a cooling effect and is used as an antipyretic, cardiotonic, laxative, antiasthmatic, and anthelmintic [10]. Bhandari et al. (2010), Rana et al. (2018), and Bhattacharjee (2013) provided local names and botanical descriptions of *P. kurroa* [11,12,13]. *P. kurroa* has been used to treat respiratory diseases, allergies, inflammatory conditions, fever, diarrhea, chronic scorpion stings, asthma, and liver-related diseases [11,14,15,16]. Hepatoprotective effects against carbon tetrachloride, alcohol, isoniazid, paracetamol, amanita mushrooms, rifampicin, *Plasmodium berghei*, and aflatoxin B1 poisoning have been reported [17,18,19,20,21,22,23]. Jaundice can be treated with *P. kurroa* [24]. The ethanolic extract contains 50–60 percent of two iridoid glycohepatosides in the ratio of 1:1.5, picroside-I, and kutkoside, which have been proven to have powerful hepatoprotective effects against miscellaneous hepatotoxins, including alcohol in preclinical studies. The active constituents of kutki consist of kutkoside and iridoid glycosides (IGs) (picroside I and II), which are primarily found in the rhizome and roots [25]. 

No major adverse effects have been reported for this plant. The diverse pharmacological activities of *P. kurroa*, a medicinally significant endangered plant, have led researchers to develop practical techniques for its in vitro mass multiplication [7,8,9]. The International Union for Conservation of Nature (IUCN) reported that this species should be protected under the rare endangered species (RET) category. Practical actions, such as in situ and ex situ conservation, are required to preserve this plant [26,27]. 

In recent years, *P. kurroa* has piqued the interest of many researchers who are working excitedly to identify the active ingredients of this plant and the mechanisms by which they exert their effects. In this comprehensive review, the phytoconstituents of *P. kurroa* were examined for their substantial pharmacological activities, such as antimicrobial, anti-inflammatory, anticancer, immunomodulatory, antidiabetic, antioxidant, hepatoprotective, anti-ulcer, cardioprotective, hypolipidemic, and others. A broad spectrum of phytoconstituents, including glycosides, cucurbitacins, iridoids, alkaloids, terpenes, and phenolics, have shown many promising pharmacological properties. Studying the various secondary metabolites of *P. kurroa* and their biological targets is necessary to determine the mechanisms of action with clinical benefits. Furthermore, this review examines the ethnopharmacological uses, biological and chemical properties, clinical evidence, and toxicology of *P. kurroa*. This information will be a source for future fundamental and clinical studies.

## 2. Ethnobotanical Properties of *P. kurroa*

**Figure 1:**
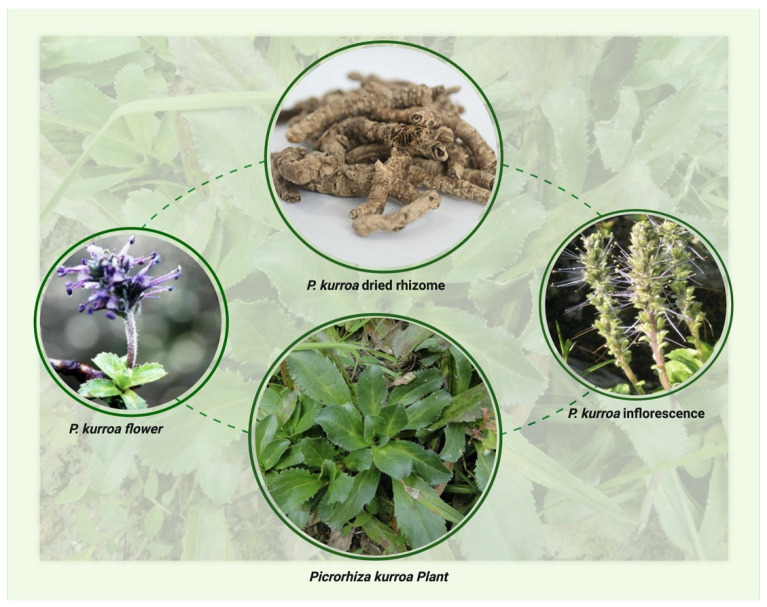
The morphological features of *Picrorhiza kurroa*.

*Picrorhiza kurroa* plants have a long creeping rootstock that is bitter and grows in rock crevices and moist, sandy soil. The plant’s leaves are oval, flat, and serrated. The flowers are pale purple or white, appear from June to August, and are borne on a tall spike. The plants are harvested manually from October to December. The morphological features of the plant are depicted in Figure 1.

Traditional medicine is affordable and widely available in developing nations, and many people rely on it for their primary healthcare needs. In Ayurveda, *P. kurroa* is known as “kutki” or “Katuka” and has several health advantages recorded in both Samhitas (medical treatise of Ayurveda) [28]. Nighantu (an important treatise on ayurvedic herbs that mentions names of drugs from various sources, such as vegetables, minerals, and animals, and their synonyms, actions, and uses) examined and discussed the qualities and dosages of *P. kurroa* for treating various illnesses [29]. Adarsa Nighantu also explains how to properly use the herb’s root portion as the recommended daily dose [30]. Tibetan medical experts and non-specialists use this plant’s rhizomes to cure colds, coughs, skin diseases, liver diseases, fever, indigestion, jaundice, scorpion stings, hepatitis, and metabolic problems [31,32]. A brief botanical description of *P. kurroa* is shown in Table 1.

## 3. Phytochemistry

**Figure 2:**
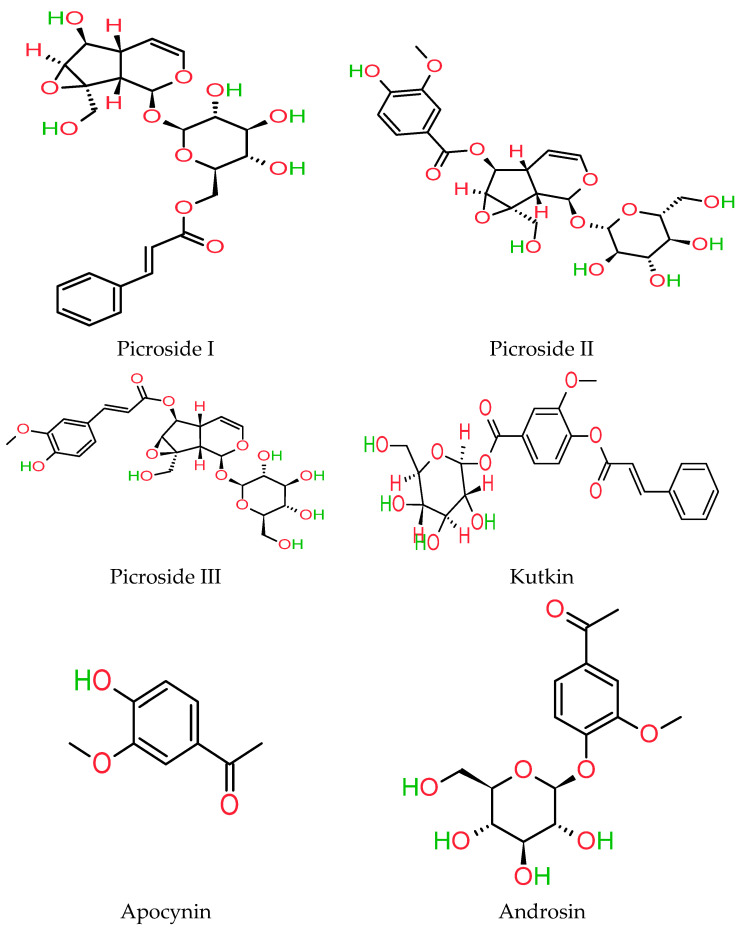

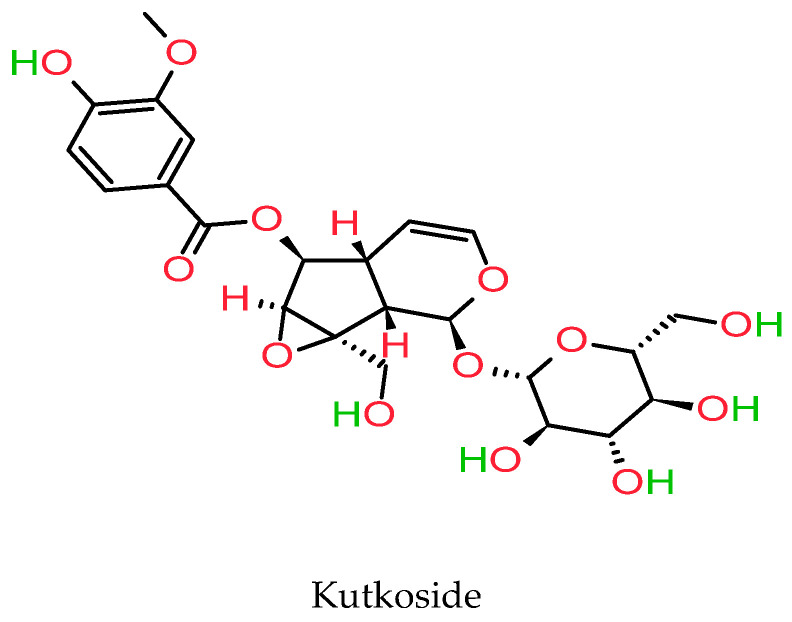
Major phytoconstituents extracted from *P. kurroa*.

*Picrorhiza* has been widely explored in terms of its chemistry, and several physiologically active compounds have been extracted from its rhizomes, roots, seeds, stems, and leaves [33,34,35,36,37]. This plant yielded more than 65 secondary metabolites. *P. kurroa* rhizomes and roots produce a crystalline substance called “Picroliv” or “Kutkin,” a combination of two C9-IGs known as kutkoside. Picroside-II and picroside-I were found in a ratio of 2:1 [38]. Picrosides I, II, and III, cucurbitacins, and kutkoside are IGs identified in the rhizomes of *P. kurroa* and determined using HPLC (high-performance liquid chromatography) techniques [39]. *P. kurroa* has various bioactive chemicals with pharmacological and therapeutic potential, including glycosides, iridoids, alkaloids, phenolics, terpenes, and cucurbitacins [40,41,42,43,44]. In addition to its purgative and emaciating properties, *picrorhiza* also has abortifacient and digestive properties, cholagogue, appetizing properties, and heart and liver-stimulating actions [45]. The active constituent of *P. kurroa*, known as kutkin, is a mixture of kutkoside and picroside. Kutkin (picrosides and kutkosides) has shown hepatoprotective properties pharmacologically. Drosin and apocyanin are two other bioactive chemicals isolated from this plant. Apocynin is a catechol that may counteract the oxidative response of neutrophils [46]. Steroidal glycosides such as cucurbitacins B, R, and D have been isolated from chloroform/ethyl acetate and methanol extracts of the roots of *P. kurroa* [30,36,47,48,49]. Cucurbitacins B, D, and R, found in *P. kurroa*, are well-known for their anti-tumorous and cytotoxic properties. Apocynin is a potent NADPH (nicotinamide adenine dinucleotide phosphate) oxidase inhibitor and has anti-inflammatory and antioxidant properties. Androsin has an antiasthmatic effect [50]. LC–ESI–MS/MS (liquid chromatography–electrospray ionization/multi-stage mass spectrometry) techniques have also reported the presence of picrosides (I, II, III, and IV), kutokoside, pikurosides, and flavonoids such as vanillic acid and apocynin in the 70 percent hydroalcoholic fraction [34]. *P. kurroa* also contains other important compounds, e.g., carbohydrate D-mannitol and aromatic acids such as vanillic acid, cinnamic acid, and ferulic acid [51,52]. Figure 2 shows various secondary metabolites of *P. kurroa*.

Some studies have performed a physicochemical examination of *P. kurroa*. The total ash (5.92% *w*/*w*), pH of 10% aqueous solution (12.95), moisture content (12.95% *v*/*w*), insoluble acid ash (2.14% *w*/*w*), water-soluble ash (3.35% w/w) were reported [53,54,55]. Vanillic acid, the main phenolic component, was calculated to be present in *P. kurroa* roots at a rate of 161.2 mg/100 g dry weight [56]. A separate investigation compared the total phenolic content of methanolic and aqueous extracts at various concentrations (50, 100, 150, and 200 g). The methanolic extract (14.11–35.36 g GAE) showed a more considerable amount of total phenolics than the aqueous extract (10.79–24.87 g GAE) [57]. Based on HPLC analysis, the total phenolic content of roots was determined to be 3.14 mg/100 g of dry weight [58]. In a related investigation, 222 μg GAE/mg and 197 μg QE/mg of dry root were determined to be the total phenolics and flavonoids in a 70% ethanol extract [34]. The hydro methanolic extract of *P. kurroa* was calculated to contain 544 mg GAE/g and 400 mg RE of total phenolics and flavonoids, respectively [59]. 

## 4. Pharmacological Activities of *P. kurroa*

**Figure 3:**
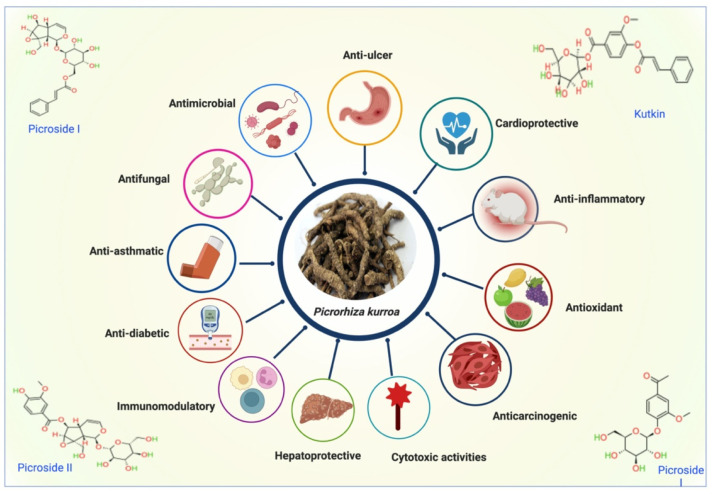
Pharmacological activities of *P. kurroa*.

The genus *Picrorhiza* contains 22 types of IGs. The seven different kinds of such glycosides as kutkoside, kutkin, pikuroside, picroside V, bartsioside, boschnaloside, and mussaenoidic, together called “iridoid glycosides” make *P. kurroa* unique. They are found in the plant’s leaves, roots, and rhizomes [26,60]. IGs are a family of natural materials used for therapeutic purposes since the dawn of time to treat various illnesses [61,62]. They have a wide range of biological applications, including antiasthmatic, and immunomodulatory [63], in treating hepatitis B, leukoderma, urinary and gastrointestinal issues, and liver illnesses, both in traditional and contemporary medicine [64,65]. *P. kurroa* rhizomes exhibit anticancer activity due to bioactive components such as IGs (picrosides I and II), apocyanin, and cucurbitacins [66,67,68,69,70]. Bis-iridoid and six IGs were isolated by Win et al. from the butanol extract of *P. kurroa* stems collected in Myanmar. These compounds have shown antiviral activity without notable cytotoxicity [71]. Kumar et al. tested the anti-inflammatory effects of *P. kurroa* rhizome extract on granuloma formation caused by cotton pellet implantation and paw edema induced by carrageenan. Research findings have demonstrated that pretreatment may suppress, in a dose-dependent manner, the development of granulomas caused by cotton pellets and paw edema caused by carrageenan. It has been established that *P. kurroa* extract may inhibit the multiplication of inflammatory cells such as macrophages, neutrophils, and mast cells. The results of this study confirmed that the anti-inflammatory effect of *P. kurroa* is mediated through the modulation of NF-κB signaling, which suppresses the production of macrophage-derived cytokines and mediators [72]. The pharmacological efficacy of *P. kurroa* is shown in Figure 3.

Phytochemical analysis of *P. kurroa Benth* rhizomes demonstrated the existence of bioactive components associated with antibacterial activities. When tested on different extracts of *P. kurroa* rhizomes, various chemical tests and TLC (thin layer chromatography) examinations revealed the presence of glycosides, sterols, and phenolic compounds. The ethanolic and methanolic extracts of *P. kurroa* have exhibited antibacterial potential against Gram-positive bacteria such as *Bacillus subtilis*, *Staphylococcus aureus*, and *Micrococcus luteus*, and Gram-negative bacteria such as *Escherichia coli* and *Pseudomonas aeruginosa* [73,74,75]. Another study found that cucurbitacins are cytotoxic and have anticancer properties [41]. In Chinese herbal medications, glycosides such as agnusides and negundoside have been used to treat various ailments, including scalds, burns, gonorrhea, cough, bacterial dysentery, colds, bronchitis, and rheumatic arthritis [76]. The economic parts of the plant are rhizomes and dried roots. Small doses of *P. kurroa* have been used as stomachic and laxatives [77]. The hepatoprotective effect of *P. kurroa* in combination with honey was investigated in a mouse model of acetaminophen administration. By reducing the activity of serum glutamic-oxaloacetic transaminase (SGOT) and serum glutamic pyruvic transaminase (SGPT) in the liver, the damage caused by acetaminophen was decreased to normal levels [78]. Another study showed that plant leaf extracts could ameliorate anemia caused by phenylhydrazine. It has been shown to help hemoglobin, red blood cell counts, immature blood cells without a nucleus, and blood volume [79]. 

Additionally, the plant has been observed to aid in repigmentation recovery in vitiligo [80]. Preclinical studies confirmed that *P. kurroa* extract at a dose of 2000 mg/kg body weight was safe in Wistar rats and might be used in standardized formulations [81]. Thus, *P. kurroa* is a valuable medicinal herb. Historically, it has been extensively employed in human health treatments. More studies are required to develop the literature on utilizing insect-pest control, organic nutrition sources, and insect-pest control measures since expanding the procedures for *P. kurroa* would be helpful. The hepatoprotective and health-promoting characteristics of *P. kurroa* may improve health and family income globally. Therefore, further clinical studies should be conducted to establish their preventive properties. Table 2 lists the numerous pharmacological characteristics of the rhizomes of *P. kurroa*.

## 5. Classical and Clinical Use of *P. kurroa*

*P. kurroa* mentions its clinical use in the ancient and classical treatise of Ayurveda-Charaka-Samhita [101]. Since the medieval period, it has been used in various clinical conditions [102]. Its clinical effectiveness is generally accepted in modern Asian medical practice, with a few restrictions [103]. For most clinical indications, an adult should consume *P. kurroa-*Kutki rhizome powder at doses between 300 mg and 500 mg two to three times a day [104]. Clinical practices with *P. kurroa* have shown that its traditional preparations exhibit adverse symptoms, such as diarrhea, increased bowel frequency, abdominal gurgling, and abdominal colic syndrome [105]. Table 3 shows clinical trials involving *P. kurroa* in combination with other agents and *P. kurroa* (alone) for liver-related and other conditions.

### 5.1. Antimicrobial Activity 

Microbial infections cause many diseases, and bacteria are the most common microorganisms that cause opportunistic diseases linked to various other diseases [115,116,117,118]. However, repeated prescriptions of antibiotics over the past three decades have led to the development of resistance against microbial infections, leading to complications in disease management [119,120]. Therefore, there is an urgent need to investigate the antimicrobial activities of *P. kurroa* to develop new drugs. Apocynin, a constituent of *P. kurroa,* is a catechol. It is a strong anti-inflammatory drug, while cucurbitacins are highly cytotoxic and antitumor in nature [36]. Rathee et al. examined the methanolic and aqueous extracts of *P. kurroa* against fungi and Gram-positive and Gram-negative bacteria. An in vitro study against two strains of fungi, *Aspergillus niger* and *Candida albicans*, and five strains of bacteria, *Escherichia coli*, *Pseudomonas aeruginosa*, *Staphylococcus aureus*, *Micrococcus luteus*, and *Bacillus subtilis*, was conducted. The MIC (minimum inhibitory concentration) values and cup plate techniques were used to examine the antimicrobial activity of *P. kurroa*. Unsaturated triterpenes/sterols, polyphenols, IGs, and cucurbitacins showed positive results. 

In an investigation, the phytoconstituents, such as phenolic compounds, glycosides, triterpenoids, and sterols, were extracted from the methanolic extract of *P. kurroa*. The methanolic extract showed significant activity against *P. aeruginosa* and *S. aureus* and moderate activity against *E. coli*, *B. subtilis*, and *M. luteus*. In contrast, the aqueous extract had no action against any tested fungi, while the methanolic extract had activity against *Candida albicans* and no activity against *Aspergillus niger* [75]. The ethanol rhizome extract of *P. kurroa* exhibited significant antibacterial activity against *B. cereus*, *S. aureus*, *K. pneumoniae*, *S. pyogenes*, *S. typhi*, and *E. coli,* while the methanol rhizome extracts showed significant antibacterial activity against *P. aeruginosa* and *S. aureus*. In contrast, hexane and acetone extracts showed moderate antibacterial activity against *S. pyogenes*, *P. aeruginosa*, *S. typhi*, *K. pneumoniae*, *E. coli*, *B. cereus*, and *S. aureus.* The antibacterial efficacy of aqueous rhizome extracts against the investigated bacterial strains was inconclusive in this study [74]. Another study examined that the methanol extract of *P. kurroa* had significant antimicrobial activity compared to ciprofloxacin, and this extract also had significant activity against fungal strains compared to fluconazole [121]. Dried stolons of this plant exhibit broad antibacterial action against various pathogenic microorganisms, including *Erwinia chrysanthemi*, *Gloeocercospora sorghi*, *Rhizoctonia solani*, *Sporisorium scitamineum*, and *Fusarium oxysporum* [122]. The alcoholic extract of the root of *P. kurroa* was examined to determine its antifungal activities. It was effective against *Trichophyton rubrum*, *Penicillium marneffi*, *Candida albicans*, and *Candida tropicalis* [85,122]. Recent studies have shown that methanol and ethanol extracts of *P. kurroa* rhizomes include compounds that might be used to develop a new broad-spectrum antibacterial formulation. The findings presented here make *P. kurroa* a good antibacterial candidate for further mechanism-based studies.

### 5.2. Anti-Inflammatory Activity

Nonsteroidal anti-inflammatory drugs (NSAIDs) treat inflammation and discomfort but have many adverse side effects. Medicinal plants have valuable anti-inflammatory properties and little or no adverse effects [118,123]. The phytochemical apocynin, which is found in root extracts of the medicinal plant *P*. *kurroa*, has been shown to have anti-inflammatory activities. The researchers examined the influence of apocynin on the synthesis of inflammatory mediators produced from arachidonic acid by pulmonary macrophages of guinea pigs. The production of thromboxane A_2_ was suppressed by apocynin in a concentration-dependent manner, but the release of prostaglandins E_2_ and F_2_ increased. In addition, apocynin significantly decreased the aggregation of bovine platelets induced by arachidonic acid, perhaps by inhibiting thromboxane production. These findings imply that apocynin, in addition to its anti-inflammatory actions when administered as a root extract of *P. kurroa*, might be an effective tool for developing novel anti-inflammatory or antithrombotic medications. Apocynin may be implicated in the anti-inflammatory activities of *P. kurroa* root extracts via mechanisms other than oxygen radical generation by inflammatory cells [124]. 

The rhizome extract of *P. kurroa* reduced inflammation in rat cotton pellet implantation-stimulated granuloma and carrageenan-induced paw edema. The results of this study showed decreased levels of inflammatory cytokines (IL-1β, IL-6, and TNF-α) and increased levels of pro-inflammatory cytokines (IL-10) in the serum and peritoneal macrophages [125]. In another study, the rhizome extract of *P. kurroa* showed an anti-inflammatory effect through the suppression of inflammatory TNF-receptor-1, cyclooxygenase-2, iNOS and inhibited NF-κB activation in lipopolysaccharide-induced RAW264.7 macrophages. This was accomplished by inhibiting the phosphorylation of IκB kinase alpha [72]. The role of IGs in anti-inflammatory actions has been shown, principally via the reduction of reactive oxygen species (ROS) and restraint of pro-inflammatory cytokines. According to current research, their anti-inflammatory effect may be attributed to the lower expression of NF-κB in renal tissue and sciatic nerve due to IG pre-treatment. Modifications in TNFα and IL-1β also demonstrated the anti-inflammatory effect of IGs pre-treatment [126]. IGs derived from *P. kurroa* can alter the expression of NF-κB in cancer cell lines H1299, KBM-5, and A293 by blocking the activation pathway [127]. The protective effect of IGs is attributed to PPAR-γ (peroxisome proliferator-activated receptor gamma) antagonism, which causes apoptosis inhibition and pro-inflammatory cytokine reduction. According to these findings, IGs derived from *P. kurroa* can potentially be beneficial in treating disorders affecting the PPAR-γ pathways [128,129]. However, further research in this area is required.

### 5.3. Antioxidant Activity

The field of preventive medicine is increasingly interested in using plant-derived antioxidants rather than synthetic ones [130,131,132]. Therefore, further exploration and characterization of natural resources are needed. It has also been observed in various studies that *P. kurroa* has antioxidant action [82,133]. The antioxidant activities of the aqueous and methanolic PKRE (*P. kurroa* rhizome) extracts were used to examine the ferric-reducing antioxidant activity, radical scavenging assay, and thiobarbituric acid assay to test the suppression of lipid peroxidation. Both extracts exhibited good antioxidant properties [57]. *P. kurroa* has shown that the plant has DNA-preserving properties [134]. The leaves of *P. kurroa* were investigated as a potential natural source of antioxidants. By using column chromatography, picein and luteolin-5-O-glucopyranoside were successfully isolated from the butanol extract. Two assays, 2,2′-azino-bis (3-ethylbenzothiazoline-6-sulphonic acid) and a 2,2-diphenyl-1-picrylhydrazyl radical, assessed the antioxidant activity of all the extracts and isolated compounds. Compared to ethanol extracts, ethyl acetate and butanol extracts have shown antioxidant activity [135]. 

In another study, DPPH (1,1-diphenyl-2-picrylhydrazyl) was used to investigate the antioxidant potential of the two *Picrorhiza* species. *P. scrophulariiflora* and *P. kurroa* have scavenging activities of 37.70 and 34.30 percent at 0.1 mg/mL, respectively [136]. IC_50_ (half-maximal inhibitory concentration) values of Viscozyme and Protamex extracts from *P. kurroa* were 1.89 and 0.46 mg/mL, respectively, for hydroxyl radical scavenging activity. The protective effect of Protamex extracts from *P. kurroa* on DNA impairment caused by free radicals was 92 percent effective at a concentration of 3 mg/mL. According to these findings, enzymatic extracts of *P. kurroa* exhibited significant antioxidant activities [137]. Furthermore, at a dose of 20 mg/kg body weight, the ethanol extract of *P. kurroa* rhizomes has been reported to scavenge free radicals in rats with gastric ulcers caused by indomethacin [133]. However, further in vitro and in vivo studies are required to understand the antioxidant action of *P. kurroa* at the cellular level.

### 5.4. Hepato-Protective Activity

The liver plays a vital role in the excretion of waste metabolites, digestion, bile synthesis, and most metabolic processes. A pathological liver affects all these essential functions, which affects an individual’s health. Due to the scarcity of safe, dependable, and affordable hepatoprotective medications, most patients with liver diseases prefer to use complementary and alternative medicines to manage and treat hepatic complications [138]. Folk and traditional medicine systems have described the use of roots and rhizomes of *P. kurroa* to protect the liver [27,139,140,141]. The powder of roots and rhizomes, decoction, infusion, confection, and alcoholic extract of the roots and rhizomes help treat liver diseases, such as chronic diarrhea, cholestasis, upper respiratory tract infections, dyspepsia, fever, as an immune modulator, and antioxidants [142].

*P. kurroa* is a successful recommendation for restoring various liver-related issues, including anorexia, nausea, jaundice, dyspepsia, viral hepatitis, and periodic fevers [143,144,145]. The *P. kurroa* extract’s principal active component for liver protection is kutkin. The hepatoprotective activity of kutkin is mainly due to the suppression of xanthine oxidase inhibitors, metal-ion chelators, oxygen anion generation, free radical scavenging, and anti-lipid peroxidation [134,146,147]. Glycosides in alcoholic extracts are more effective than pure functional compounds in treating live ailments [148]. In a study, male rats were administered CdCl_2_ (0.5 mg/kg, sc) 5 days/week for 18 weeks to test picroliv’s therapeutic efficiency. Picroliv was orally administered at 6 and 12 mg/kg dosages to the group that had been receiving cadmium (Cd) for the last four weeks. Cd-induced liver functional parameters, such as γ-glutamyl transpeptidase (GGT), lactate dehydrogenase (LDH), alanine aminotransferase (ALT), aspartate aminotransferase (AST), and alkaline phosphatase (ALP) levels, were reduced by picroliv. This study confirmed that picroliv has hepatoprotective and renal protective activities [149]. Another study was conducted to investigate whether freeze-dried methanolic extracts of *Berberis lycium* root bark and *P. kurroa* rhizomes provided any degree of hepatoprotection against lantadenes-induced sub-chronic hepatopathy in guinea pigs animal models. A 200 mg/kg body weight dose of *B. lycium* and *P. kurroa* extracts reduced the hepatotoxicity caused by subchronic exposure to lantadenes [150]. 

**Figure 4:**
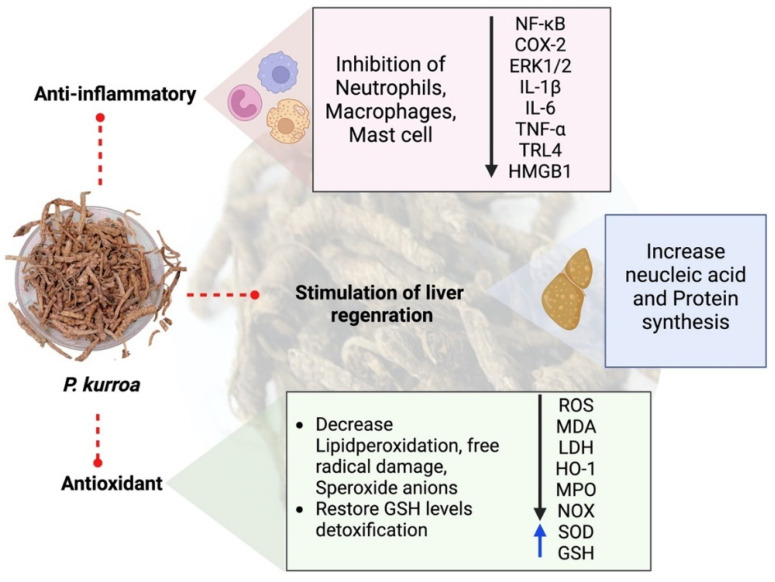
The possible mechanism of action of anti-inflammatory, antioxidant, and hepatoprotective activity of *P. kurroa*.

Furthermore, the effects of picroliv and IG combinations from the roots and rhizomes of *P. kurroa* on ethanol-induced toxicity in isolated rat hepatocytes were examined to test the GPT, GOT ALP, and aldehyde dehydrogenase in hepatocytes. Studies have shown that picroliv has a preventive effect on alcohol-induced hepatotoxicity [151]. A study in rats showed the hepatoprotective activity of picroliv and standard silymarin against AFB1 (aflatoxin B1) toxicity in the kidney and liver [141]. Saraswat et al. investigated ethanol-induced toxicity in rats and found a dose-dependent hepatoprotective action [152]. The effects of the ethanolic extract of *P. kurroa* roots and rhizomes were examined against rifampicin- and isoniazid-induced hepatitis in rats. Outcomes have shown that this extract reduces drug-induced transformation in rats and retains their normalcy [18,153]. In another study, three rat models were used to examine the toxicity induced by carbon tetrachloride (CCl_4_) and *Entameba histolytica*. These findings demonstrated a substantial recovery in the blood enzyme levels of all rats used as a model for amoebic liver abscess in gerbils after treatment with picroliv. Therefore, it can be concluded that picroliv has therapeutic effectiveness against *E. histolytica* and stimulates hepatic damage [154]. Moreover, *P. kurroa* extract showed promising benefits against lantadenes-induced hepatotoxicity and significantly reverted these abnormalities to a state close to normal [150]. A herbal preparation known as Enliv containing *P. kurroa* elevated lipid peroxidation levels in liver tissues and reduced the level of glutathione induced by paracetamol [155]. Kutkin was more effective than standard silibinin against toxic doses of *Amanita mushrooms* [155]. Preclinical research has confirmed that picroliv reduces ethanol-induced hepatotoxicity in rats [27,152]. Although very little research has been conducted on picroliv hepatoprotective characteristics, several concerns must be solved. First, the potentially harmful effects of picroliv should also be investigated at larger doses and when used for longer. It is necessary to examine the effectiveness of picroliv against various toxins and establish their safety. This should be the focus of future research. These studies confirmed that picroliv is helpful in liver-related disorders and may open new avenues for clinical trials. The possible mechanism of action anti-inflammatory, antioxidant, and hepatoprotective activities of *P. kurroa* are shown in Figure 4. 

### 5.5. Antidiabetic Activity

The development of hypoglycemic medications, diabetes, and its associated consequences continue to be a significant concern in medicine. Diabetes can be managed with the help of several medicinal plants [156,157]. A study found that *P. kurroa* extract could eliminate oxygen free radicals such as superoxides and hydroxyl radicals and stop lipid peroxidation in rat liver homogenate caused by the Fe^2+^ ascorbate system [158]. It has been recognized that alloxan exerts its diabetogenic action primarily by producing oxygen-free radicals, thereby harming the pancreas [159]. Serum lipid peroxide levels were reduced in alloxan-induced diabetic rats by *P. kurroa* and blood urea nitrogen while inhibiting weight loss and leukopenia. These findings suggest that *P. kurroa* extracts may mitigate the metabolic damage caused by alloxan in diabetic rats [86]. In a study, streptozotocin- and nicotinamides-induced diabetic rats model provided in vivo evidence that a standardized extract of *P. kurroa* has considerable antidiabetic action in rats with type-2 diabetes mellitus [87]. Another study evaluated the mechanism of antidiabetic activity of a standardized aqueous *P. kurroa* extract to streptozotocin-induced diabetic rats for 14 consecutive days. It was shown that *P. kurroa* extract boosted the insulin-mediated transfer of glucose absorption from the cytosol to the plasma membrane, which increased the skeletal muscle’s ability to take up glucose [88]. Kumar et al. examined the effect of hydro-alcoholic extract of *P. kurroa* rhizome (PKRE) in insulin-producing Rin5f cells and streptozotocin (STZ)-induced diabetic mice. PKRE was shown to contain over 30 metabolites, including picrosides I and II, as determined by ^1^H-NMR spectroscopy. According to the findings of this study, treatment with PKRE at dosages of 100 and 200 mg/kg for 30 days significantly protected pancreatic β-cells from STZ-induced damage [160]. *P. kurroa* is a hepatoprotective agent; therefore, increased blood glucose utilization and absorption might be the additional activity of the extract. Therefore, it is necessary to confirm the antidiabetic mechanism of action of *P. kurroa* by examining insulin levels and receptors. 

### 5.6. Anticancer Activity

**Figure 5:**
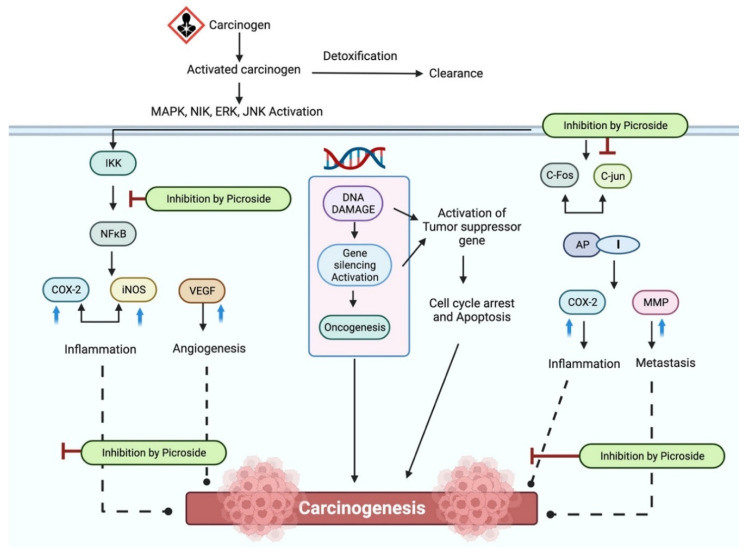
The probable anticancer mechanism of action of *P. kurroa*.

Cancer is the second leading cause of death worldwide, and it is anticipated that during the next few years, it will become the top cause of death [161,162,163]. Investigators have been looking at possible biological molecules to treat cancer as alternative treatment options [164,165,166]. *P. kurroa* extracts and their components may protect against cancer through many cellular and molecular processes in various disorders. Flavonoids such as apocynin, found in the rhizome, are thought to be responsible for the plant’s anticancer properties [36,66,67,68,69,167,168]. A study examined the antineoplastic and antioxidant properties of methanolic and aqueous extracts of *P. kurroa* rhizomes. The cytotoxicity of the extracts was examined using the XTT (cell proliferation kit II) assay in human breast carcinoma (MDA-MB-435S), human hepatocellular carcinoma (Hep3B), and human prostate cancer (PC-3). It was observed that all three cell lines examined were susceptible to apoptosis induction by methanolic and aqueous *P. kurroa* extracts [57]. Another study outcomes strongly recommend that picroliv is a favorable agent for alleviating injury following chemotherapy and radiation [169]. Plant extracts have shown cytotoxic and antioxidant properties that could be helpful in metabolism to fight cancer. In mice, the anticancer activity of picroliv was shown to be stimulated by N-nitrosodiethylamine, which led to the development of hepatocellular carcinoma [99]. In vitro and in vivo studies confirmed that PII efficiently reduced the metastasis and angiogenesis of cancer cells. Therefore, it may be an innovative option for cancer treatment [170]. However, further research is needed to discover specific action methods and to investigate signal transduction pathways to understand biological processes better. The probable anticancer mechanism of action of *P. kurroa* is shown in Figure 5.

### 5.7. Immunomodulatory

Immunomodulatory plants, via their effects on numerous cell types through interleukins and cytokines, play an essential role in treating inflammation [171,172], infection [173], and immunodeficiencies [174]. Immunosuppressants, immunoadjuvants, and immunostimulatory agents can boost the immune response to antigens [174,175]. Immunosuppressants may help relieve various autoimmune and hypersensitivity diseases, in contrast to immunostimulants used to treat cancer [176]. A study investigated the effects of the biopolymeric fraction RLJ-NE-205 from *P. kurroa* on in vivo immunological function in mice. The results showed a significant increase in cytokine levels (IFN-γ and IL-4) and lymphocyte proliferation at a dose of 50 mg/kg, which may be considered a biological response modulator [90]. Another study showed that a 50% ethanolic extract of *P. kurroa* leaves enhances cell-mediated and humoral immune components and phagocytosis in experimental animals. In addition, the extract of *P. kurroa* leaves improved both the humoral immune response in rats and mice and the phagocytic activity of cells in the reticuloendothelial system in mice [91]. Additionally, the immunomodulatory effects of ethanolic extracts of *W. somnifera*, *A. racemosus*, and *P. kurroa* were investigated. These three herbs have shown immunostimulatory effects in the order of *W. somnifera* > *A. racemosus* > *P. kurroa* [92]. In conclusion, all discussed studies have shown the immunostimulatory activity of *P. kurroa*, suggesting its therapeutic usefulness.

### 5.8. Anti-Ulcer Activity

Despite recent pharmacological advances, gastrointestinal toxicity remains a primary medical concern due to gastrointestinal toxicity associated with NSAID drugs [177,178]. The gastroprotective effects of *P. kurroa* have been examined in various animal studies. *Picrorhiza* is regarded as a liver tropho restorative herb and robust immune booster [179]. It has also been claimed that picroliv exerts choleretic action [180]. Indomethacin-induced stomach ulcers in mice were efficiently healed by the methanol extract of *P. kurroa* rhizomes. This is accomplished by promoting mucin secretion, lowering oxidative stress, increasing prostaglandin production, and elevating the expression of cyclooxygenase enzymes [181]. The anti-ulcerogenic effects of the ethanol extract of *P. kurroa* roots and rhizomes on ethanol/HCl-induced ulceration in rats were investigated against pepsin, antioxidant status, glycoproteins, and proteins in the gastric mucosa. The results showed that oral treatment with *P. kurroa* roots and rhizomes for ten days significantly reduced the stimulated ulceration [182]. The anti-ulcer effect of this plant is due to vascular endothelial growth, augmented mucus production, COX-2 (Cyclooxygenase-2), SOD (superoxide dismutase), EGF (epidermal growth factor), and PG (prostaglandin) synthesis, and reduced LPO (lipid peroxidation) stress mediators such as TBARS (thiobarbituric acid reactive species) [133].

### 5.9. Cardioprotective Activity

Therapy for cardiovascular problems often involves using compounds extracted from various plant species. For example, *P. kurroa* is an astringent medication abundant in IGs and participates in different pharmacological processes [71]. *P. kurroa* has been shown to have cardioprotective benefits against stress caused by isoproterenol in the myocardium of rats [183]. *P. kurroa* (200 mg/kg) did not significantly change in pre-treated normal rats during isoproterenol administration, resulting in left ventricular dysfunction, hemodynamic changes, lipid peroxidation, and oxidative stress. However, the restoration of catalase, glutathione peroxidase enzymes, and myocardial superoxide dismutase, except for decreased glutathione levels, was one of the primary mechanisms by which pretreatment dramatically mitigated isoproterenol-induced oxidative stress. The *P. kurroa* root extract exhibited a potent cardioprotective effect based on these findings. This effect may be attributable to the anti-peroxidative, antioxidant, and myocardial preservation markers contained in the extract [184]. A study in rats has shown that *P. kurroa* has cardioprotective properties against isoproterenol-induced myocardial stress. Oral treatment with *P. kurroa* significantly stopped the myocardial infarction caused by isoproterenol and kept the rats’ health close to normal [185]. *P. kurroa* exhibits cardioprotective activity due to its free radical-scavenging property [147]. The present section stated that the therapeutic and preventive potential of *P. kurroa* phytoconstituents for controlling cardiovascular illnesses had been examined using numerous methods; however, the molecular processes are still unknown.

### 5.10. Hypolipidemic Effect

The presence of high levels of cholesterol in the blood is one of the most critical risk factors for the development and progression of coronary heart disease [186]. Hyperlipidemia is also induced by the secondary effects of diabetes [187]. Hyperlipidemia is a risk factor for liver disease [188]. Lee et al. evaluated the hepatoprotective and hypolipemic potential of PKRE extracts in high-fat diet-hyperlipidemic mice. Total cholesterol, LDL, TGs (triglycerides), liver weight, ALT, and AST levels were significantly decreased by this treatment. On the other hand, *P. kurroa* water extract did not seem to affect serum HDL levels. The study outcomes showed that PR water extracts had a reasonably excellent and beneficial impact on high-fat diet-induced hyperlipidemic mice with good hepatoprotective properties [189]. The hepatoprotective properties of the water extract were also found to have a preventive hypolipemic effect in another study of PR in mice that had developed hyperlipemia by poloxamer (PX)-407. The effectiveness of PX-407 was compared with that of 10 mg/kg simvastatin. Serum TGs, LDL, and total cholesterol levels were reduced dose-dependent in the PR extract and simvastatin dosing groups compared to the vehicle control group. These findings suggest that the PR water extract has a comparatively excellent positive preventative effect on PX-407-induced hyperlipidemia and a favorable hepatoprotective impact [190]. As a result, it is reasonable to anticipate that the PR extract has positive potential for developing hypolipemic and hepatoprotective medicines.

### 5.11. Antiasthmatic Activity

One significant cause of mortality is asthmatic inflammation, which is mediated by the Th_2_ cytokine response [191]. It is a chronic inflammatory ailment of the airways, characterized by variable airflow constraints and airway hyper-responsiveness to various stimuli [192]. Phenol glycoside and rosin have been identified as active components that prevent allergen and platelet-activating factor-induced bronchial obstruction in guinea pigs exposed to inhalation challenges [193]. Picroliv inhibited passive cutaneous anaphylaxis in mice and rats and protected mast cells from degranulation in a concentration-dependent manner. There was a decrease in the Schultz–Dale response in the sensitized guinea pig ileum; however, the bronchospasm caused by histamine could not be antagonized or avoided by picroliv, demonstrating the lack of a direct-synaptic histamine receptor-blocking effect [194].

## 6. Dosages and Toxicity

The components of *Picrorhiza* do not dissolve in water instantly; therefore, they cannot be consumed as a tea. Generally, it is consumed in capsules containing standardized kutkin (4% kutkin). Adults should be administered 400–1500 mg/day; however, doses of up to 3.5 gm/day are occasionally prescribed for treating fevers. Antiperiodic doses of 3–4 gm have been suggested; 0.6–1.2 gm have been used as an astringent tonic. Despite their widespread use in India, there have been no documented cases of *Picrorhiza* root extracts causing adverse effects. Herbal drugs may interact with one another or with conventional pharmaceuticals, necessitating consultation with a healthcare practitioner before dose adjustment. Medicinal plants may have side effects or possess toxic elements; however, adverse effects and toxicity are commonly less prevalent than synthetic medications. All relevant warnings and contraindications should be fully understood. When purchasing medical herbs, it is vital to conduct thorough research, choose a vendor with a good reputation, and carefully read the label to ensure that you are aware of any content, instructions, or precautions [195]. 

The highest dose of a test sample that can be safely administered to rats over one day in the form of two fractional doses is less than 2000 mg/kg of body weight. The study showed that *P. kurroa* could be stored up to a temperature of 50 °C for three months, and the properties of the plant did not affect it, while the high temperature led to significant degradation [24]. Researchers investigated the oral toxicity of PKRE. Wistar rats were administered a single high extract dose of 2000 mg/kg body weight. Viability, mortality, and clinical signs were reported on test day 0 (before administration) and 7 and 14 days after death. All animals appeared healthy from the first day of the trial until completion. The extract was non-toxic to rats, and it helped them gain weight with an LD_50_ > 2000 mg/kg. No toxicologically significant side effects were associated with the oral administration of *P. kurroa*; preclinical results from this study might support the start of a clinical trial using the plant’s standardized extracts [81]. 

A study was conducted to determine whether *P. kurroa* could cause reverse mutations at the histidine locus in different strains of *Salmonella typhimurium*, with or without an exogenous metabolic activation system (S9) with microsomal enzymes. Researchers have shown that the bacterial reverse mutation assay is an excellent method to test the ability of different chemical classes to cause mutations. All the bacterial strains exhibited adverse reactions over the entire dosing range. In each of the separate studies, there was no evidence of a statistically significant dose-related increase in revertants. The *Salmonella typhimurium* reverse mutation findings have shown that *P. kurroa* rhizome extract is not mutagenic [196]. The *P. kurroa* extract at 200 mg/kg produced favorable effects against lantadenes-induced hepatic toxicity and significantly reversed these changes to near normal. The hepatotoxicity brought on by sub-chronic exposure to lantadenes may be reduced by *P. kurroa* extract at a dose of 200 mg/kg [150]. *P. kurroa* is abortifacient and should be contraindicated during pregnancy [45]. *P. kurroa* has a variety of applications; however, only a few toxicity studies have been reported. Therefore, further toxicological investigations are required to explore the various adverse effects. These investigations are essential for the handling, producing, and using of the bioactive extracts and secondary metabolites of *P. kurroa*.

## 7. Conclusions and Future Recommendations

Kutkin, kutkoside, and IGs (picroside I and picroside II) of *P. kurroa* are used in more than 2000 herbal formulations to treat various ailments. Many individuals in underdeveloped nations rely on *P. kurroa* extract to treat various human diseases. Picrosides have antioxidant properties that help cure hepatic and respiratory illnesses. In addition, picrosides exhibit different anticancer activities, such as metal ion chelation, free radical scavenging activity, cell cycle regulation, apoptotic induction, and detoxifying activity. Therefore, it has the potential to become an effective anticancer treatment in the future. Picrosides are crucial phytoconstituents, and their activity in the NF-κB pathway and future recommendations on anticancer mechanisms should be made. Since picrosides operate on a wide variety of molecular targets, the specific mechanism by which they exert their anticancer activity is not yet fully understood. The roots and rhizomes contain the phytoconstituents of this plant. *P. kurroa* is considered cathartic when consumed in high quantities and cholagogue, stomachic, and laxative when consumed in smaller amounts. 

According to preclinical investigations, oral consumption of *P. kurroa* extract at a dose of 2000 mg/kg body weight had no toxicological effects in Wistar rats. It was confirmed to be safe for use as a standardized formulation. On the other hand, biochemical research and the natural composition of *P. kurroa* have received much attention, although further studies are needed to confirm its usefulness in treating different ailments. It is necessary to study more secondary metabolites of *P. kurroa* and to identify their biological targets and mechanisms of action to treat various diseases. Additional research should be focused on confirming the synergy between the toxicity and effectiveness of using its constituents to form different formulations. Extensive in vitro and in vivo experiments and studies have been conducted to confirm the pharmacological significance of the active secondary metabolites of this herb, but there is a lack of human clinical trials to validate in vitro and in vivo studies; therefore, there is a need for more human clinical trials. We anticipate that the findings of these future investigations will serve as a foundation for new developments and applications of *P. kurroa*.

## Figures and Tables

**Table 1:** A brief botanical description of *Picrorhiza kurroa*.

Kingdom	Plantae
Subkingdom	Tracheobionta
Super-division	Spermatophyta
Division	Magnoliophyta
Class	Mannoliopsida
Subclass	Asteridae
Order	Lamiales
Old Family	Scrophulariaceae
New Family	Plantaginaceae
Genus	Picrorhiza
Species	*P. kurroa*

**Table 2:** The numerous pharmacological characteristics of the rhizomes of *P. kurroa*.

Pharmacological Activity	Action	Concentration	Study Model	Reference
Cytoprotection	Suppression of adriamycin-stimulated cardiomyopathy	50 mg/kg body weight	Rats	[82]
Anti-HBsAg activity	Hepatoprotective	-	Serum	[83]
Anti-anaphylaxis	Inhibition of passive cutaneous anaphylaxis	25 mg/kg p.o.	Mice/Rats/Guinea pig	[84]
Antileishmanial	Improved lymphocyte proliferation and antileishmanial efficacy	20–5 mg/kg dose for 12 days and 10 mg/kg for further experiments	Leishmania donovani/hamster	[84]
Antifungal Activity	Inhibition of the dermatophytic fungi	5% and 10% alcoholic solvent extracts of root and rhizome	Solidified agar plates	[85]
Anti-diabetic	lower blood glucose levels in basal requirements and a heavy glucose load level.	75 mg/kg extract of the body weight	Rats	[86]
Anti-diabetic	Non-diabetic normal rats were also subjected to an oral glucose tolerance test.	100 mg/kg–200 mg/kg, p.o	Rats	[87]
Anti-diabetic	GLUT-4 concentration over the whole of the soleus muscle’s membrane fractions	100 mg/kg/day–200 mg/kg/day	Rats	[88]
Anticancer	Protection against hypoxic injury	-	Hep3B and glioma cells	[89]
Immunomodulatory	Enhance levels of cytokines (IFN-γ and IL-4) and the lymphocytes’ proliferation	12.5 mg/kg, 25 mg/kg, and 50 mg/kg body weight for 14 days	Balb/c mice	[90]
Immunomodulatory	Enhances the cell-mediated and humoral immune components	-	Mice and rats	[91]
Immunomodulatory	Hypersensitivity reaction	100 mg/kg of body weight	albino mice	[92]
Anticancer	Highest cytotoxicity	50 mg/kg for ten days	EAC (Ehrlich ascites carcinoma tumor-bearing mice	[93]
Anticancer	Induction of cell toxicity and decrease in matrix metalloproteinases 1 and 13; 2 and 9.	50 μg/mL and 100 μg/mL	MCF-7 cell lines (Human breast cancer)	[94]
Antioxidant and anti-neoplastic	DPPH radical dot and radical dotOH, ferric reducing antioxidant activity, and thiobarbituric acid assay for testing suppression of lipid peroxidation	-	Hep3B, PC-3, MDA-MB-435S	[57]
Anticancer	FMuLv induced erythroleukemia	-	BALB/c mice	[95]
Anticancer	DMH-induced hepatic carcinogenic response	40 and 200 mg/kg	Rats	[96]
Antitumor and anti-carcinogenic	Yeast topoisomerase I and II enzyme	250 μg/mL	The span of ascites tumor bearing mice	[97]
Anticarcinogenic	Serum and tissues of tumor-bearing animals	150 mg/kg–750 mg/kg body weight	Rats	[98]
Anticancer	Hepatocarcinogenesis induced by N-Nitrosodiethylamine	200 mg/Kg body weight	Rats	[99]
Anticancer	20-methylchlanthrene- induced sarcoma model and 7,12-dimethylbenz[a]anthracene-initiated papilloma formation	100 mg/kg–200 mg/kg, p.o	BALB/c mice	[100]
Hepatoprotective	Prevention of biochemical changes in the liver and serum of galactosamine-intoxicated	12 mg/kg/day for 7 days	Rats	[16]

**Table 3:** Clinical trials of *P. kurroa* (alone) or in combination with other agents against liver-related problems and various other conditions.

Formulation	Study Design	Duration of the Study	Participants	Dose	Adverse Effect	Activity	Reference
Kutaki processed in Guduchi with Atorvastatin	Open Label	3 months	32	Atorvastatin 20 mg twice daily + 2 gm Katukai (*P. Kurroa*) processed in Guduchi twice daily	No adverse effect	Hepatoprotective	[106]
Elastographic liver evaluation of Katukyadi churna in the management of Non-Alcoholic Steatohepatitis (NASH)	A single-arm clinical trial	180	11	6 gm (Sachets) twice a day with water twice a day with water, Katukyadichurna comprises 1 part each of Katuki þ Nimba þ Amrita þ Bhringaraj þ Bhumyamalki	Two patients suffered from loose stools 2–3 times/day for the first 8 days.	NASH	[107]
Phalatrikadi Kwath	Open Label	6 months	59	40 mL × 2	No adverse effect	HbsAg + ve	[108]
Arogyavardhini and Triphala Guggulu and Pathya	Two arms	3 months	21	250 mg × 2	No adverse effect	Non-Alcoholic Fatty Liver Disease	[109]
Kutaki with Sita	Two arms	3 weeks	30 (Divided into two groups of 15)	0 ne gm × 2	No adverse effect	Amlapitta	[110]
Kutaki+ Methoxasalen	Oral and Topical	3 months	30	200 mg × 2 20 mg once	No adverse effect	Vitiligo	[111]
Kutaki	Double Blind	2 weeks	15	375 mg × 3	No adverse effect	Acute Viral Hepatitis	[112]
Arogyavardhini	Double Blind	2 weeks	20	750 mg × 3	No adverse effect	Acute Viral Hepatitis	[113]
Arogyavardhini and Punarnavadi	Open label	3 weeks	24	500 mg × 3 and 30 mL × 2	No adverse effect	Acute Viral Hepatitis	[114]

## Data Availability

Not applicable.
